# Oxidative stress-related biomarkers in Parkinson’s disease: A systematic review and meta-analysis

**Published:** 2018-07-06

**Authors:** Zeba Khan, Sharique Athar Ali

**Affiliations:** Department of Biotechnology, Saifia Science College, Bhopal, India

**Keywords:** Oxidative Stress, Biomarkers, Parkinson’s Disease, Review, Meta-Analysis

## Abstract

Parkinson’s disease (PD) is a neurodegenerative disease characterized with the loss of dopamine-producing neurons in a mid-brain. This loss is believed to be associated with number of environmental and genetic factors. Oxidative stress is found to be one of the factors responsible for the initiation and progression of PD. However, studies are still continued to confirm the connection and mechanism associated with oxidative stress and PD. This systematic review and meta-analysis aimed to assess the association between oxidative stress markers and PD, and explore factors that may elucidate the contradictions in these results. As per Preferred Reporting Items for Systematic Reviews and Meta-Analyses (PRISMA) guideline systematic literature search was carried out. Meta-analysis was carried out on pooled standardized mean differences with 95% confidence interval (CI) of patients with PD and controls using random effect model in comprehensive meta-analysis statistical software. Total 17 studies were included into which 25 oxidative stress markers were analyzed. The results revealed that oxidative stress markers [nitrate and nitric oxide (NO)] and antioxidant markers [total antioxidant status (TAS) and thiols] were not statistically different between the PD and control group (P > 0.05). In case of oxidative stress markers, levels of malondialdehyde (MDA), 8-Oxo-2'-deoxyguanosine (8-oxo-dG), and lipid hydro-peroxide (LPO) were found to be high in patients with PD as compared to controls with P < 0.05, whereas lower levels of antioxidant activity of superoxide dismutase (SOD), glucose 6 phosphate dehydrogenase (G6PD), catalase (CAT), and glutathione peroxidase (GPx) were noticed in the PD group as compared to controls (P < 0.05 for all). From the results, it is concluded that patients with PD have high oxidative stress and lower antioxidant activity, and these studied biomarkers would be used as potential diagnostic tool to measure oxidative stress in patients with PD.

## Introduction

Selective neurons hold the neurotransmitter dopamine (DA), and loss of these neurons in substantianigra pars compacta of midbrain area is linked with Parkinson’s disease (PD). Degeneration of these neurons leads to incapacitating symptoms including resting tremor, bradykinesia, muscular rigidity, and postural imbalance.^1^ Most of the cases with PD (90-95 percent) are idiopathic, and the rest are genetic. The causes of idiopathic PD include occupational or excessive exposure of pesticides, organic solvents, carbon monoxide, and some plants-derived toxins. Some studies also have that reported bacterial and viral infection may also be the one of the cause of idiopathic PD.^2^ Cellular senescence that happens due to aging is considered an apparent factor associated with the onset of PD.^3^ In case of genetic PD, number of genes has been reported that are responsible for degeneration of the DAergic neurons.^4^

From the literature it is revealed that elevated levels of free radicals [reactive oxygen species (ROS)], protein and lipid oxidation, DNA damage and reduced activities of superoxide dismutase (SOD), catalase (CAT), glutathione (GSH), etc. makes DAergic neurons of patients with PD more prone to oxidative stress (OS).^5^^,^^6^ This respective increased and decreased activity in neurons of substantia nigra parscompacta of patients with PD leads to oxidative stress which ultimately leads to neuroinflammation. There are two underlying mechanism responsible for creating oxidative stress in DAergic neurons; one is enzymes tyrosine hydroxylase and monoamine oxidase of ROS pathway, which are responsible to make DAergic neurons prone to oxidative damage, and second is fenton reaction which is carried out in nigral DAergic neurons because of the presence of iron in neurons, which further increases oxidative stress via production of superoxide radicals and hydrogen peroxide.^7^^,^^8^

Number of oxidative stress biomarkers has been reported to measure oxidative and antioxidant levels in cells using different techniques e.g. spectrophotometry, enzyme-linked immunosorbent assay (ELISA), flow cytometry, etc. however there is variation in results due to which no suitable/validated biomarker of oxidative stress has been reported in PD. Thus, this study aimed to compile all the studies that reported the oxidative stress in PD, and find out the association between oxidative stress and the PD, if any. Simultaneously, from the meta-analysis, tried to find out that studied biomarkers could be served for diagnosis/prognosis purpose in patients with PD in future.

## Materials and Methods

***Search Strategy:*** All the relevant studies were searched in PubMed, Google Scholar, Medline, Cocharane Library EMBASE, and ISI Web of Science. The searching keywords included “oxidative stress”, “Parkinson’s disease”, and “biomarkers”. Medical subject heading (MeSH) terms and free text words were used in research equations with ‘OR’ and ‘AND’ Boolean operators. References in published studies were also searched for related studies. Referencing and collection of studies was done by Zotero (version 5.0.27, Corporation for Digital Scholarship and the Roy Rosenzweig Center for History and New Media, Fairfax, VA, USA) followed by preferred Reporting Items for Systematic Reviews and Meta-Analyses (PRISMA) guidelines. All searches were conducted prior to December 2017.

***Study Selection and Data Extraction:*** Relevant studies were selected followed by strict inclusion criteria. Five inclusion criteria were there, all the studies should be case-control, results should be presented in mean with standard deviation (SD), study should be reported in peer-reviewed journal, Hoehn and Yahr^9^ scale should be used as the diagnostic criteria of PD, and samples should be descripted accurately (e.g. diagnostic criteria, sample source, and sample number). From the included studies, study design, location where study were conducted, year, age, sample size of each group, and biomarkers values were recorded. Papers were excluded if the abstract was insufficient for biomarkers values, and full text was not available. 

***Statistical analysis:*** The statistical analysis was performed using Comprehensive Meta-Analysis Software (CMA, Biostat, USA). Standard mean differences (SMD) values with 95% of confidence interval (CI) of included biomarkers of patients and controls were recorded to construct forest plot. A random-effect model over fixed effect model was used as age, sex, and ethnicity varies among studies, and studies were weighted by the generic inverse variance method (Q statistic: P < 0.10, I^2^ > 50%). I^2^ statistic was assessed for heterogeneity between studies, which described the percentage of total variation across all studies due to heterogeneity rather than to chance. P < 0.05 was considered statistically significant. For publication bias, Funnel plot was used. To check the strength of each biomarker, one-out sensitivity analysis was also performed.

***Ethical approval: ***An ethical approval was not required for this study, as it was based on data/information retrieved from published studies already available in the public domain.

## Results

**Figure 1:**
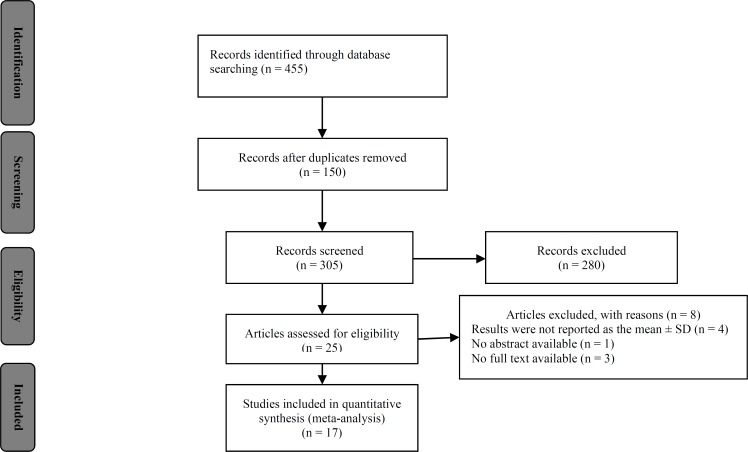
Search strategy according to Preferred Reporting Items for Systematic Reviews and Meta-Analyses (PRISMA) guideline flow diagram

***Overview of studies:*** Figure 1 shows the articles search and retrieval steps as per PRISMA flow diagram. Overall, 455 studies were retrieved from different databases and stored in Zotero software. All the studies were reviewed, and duplicates and irrelevant studies were excluded. After exclusion, 25 articles were retrieved for detailed analysis. Out of the 25 studies, 17 met the inclusion criteria for addition to the review.^10^^-^^26^ Included studies were conducted in 9 different countries. Maximum studies were conducted in an Asia continent. 10 studies were conducted in India,^12^^,^^15^^-^^17^^,^^19^^,^^20^^,^^22^^-^^25^ and the others were from Taiwan,^25^ Romania,^18^ Bulgaria,^11^ Spain,^14^ Germany,^10^ Brazil,^13^ and Turkey.^21^ Both male and female sexes were included in all studies. The age of patients and controls was in the range of 40 to 80 years. The number of cases with PD ranging from 15 to 240, and the number of controls from 15 to 150.

Biomarkers were analyzed in two categories, one was oxidative stress-related biomarkers, and the other was antioxidants-related biomarkers of PD. The association of oxidative stress as well as anti-oxidative stress-related biomarkers and the risk of PD are shown in tables 2 and 3. Percentage of inconsistency (I^2^) was found to be greater than 50% within all studied biomarkers. A high degree of inconsistency was found in 8-oxo-dG (6.018, I^2^ = 98.1%, 95% CI: 3.664-8.373) and low in nitrate (0.934, I^2^ = 32.7%, 95% CI: -0.906-2.775).

11 oxidative stress markers were studied in this meta-analysis. Out of 11 oxidative stress markers, 5 were oxidative stress-related and 6 were antioxidant-related biomarkers.

**Table 1:** The characteristics of included studies

**Authors, Country**	**Study ** **design**	**Sample ** **(patients/** **control)**	**Age (patients/control)**	**Biomarkers studied**
Oli, et al.,^10^ Germany	CS	17/12	-	8-oxo-dG
Nikolova and Mancheva,^11^ Bulgaria	CS	18/20	55-70/50-65	MDA, Protein carbonyl content, 8- oxo-dG
Nikam, et al.,^12^ India	CS	40/40	40-80/40-80	LPO, NO, SOD, GPx, CAT, Ceruloplasmin
de Farias, et al.,^13^ Brazil	CS	56/54	70.3/69.7	CL-LOOH, FOX-LOOH, MDA, TRAP, Thiols, CAT, SOD, Paraoxonase 1
Agil, et al.,^14^ Spain	CS	52/40	-	LPO, TAS
Sanyal, et al.,^15^ India	CS	80/80	-	MDA
Naduthota, et al.,^16^ India	CS	72/72	51.3 ± 10.6/51.3 ± 10.6	MDA
Vinish, et al.,^17^ India	CS	15/10	-	MDA, SOD, GPx, NO
Graciun, et al.,^18^ Romania	CS	18/16	60.8 ± 8.3/56.8±8.5	SOD, GPx, TAS, plasma TAS, G6PD, Ery MDA, Ery GPx, Ery- GSH, EryCAT, Ery-SOD
Sanyal, et al.,^19^ India	CS	80/80	58.2 ± 12.2/57.6 ± 9.1	Nitrate
Kouti, et al.,^20^ India	CS	58/15	64.4 ± 11.1/64.4 ± 11.1	Nitrate, Proxynitrite
Tuncel, et al.,^21^ Turkey	CS	25/25	67.9 ± 9.4/64.3 ± 8.0	NO
Abraham, et al.,^22^ India	CS	115/37	58.2± 0.66, 57.17± 11.21	SOD, CAT, GPx, G6PD
Sudha, et al.,^23^ India	CS	15/50	40-60/40-60	GSH, GPx, SOD, CAT
Verma, et al.,^24^ India	CS	240/150	56.4 ± 8.9/56.8 ± 11.4	Plasma prolidase, TOS, TAS, OSI
Lin, et al.,^25^ Taiwan	CS	27/25	54.6 ± 9.3/50.9 ± 10.5	TBARS, Thiols
Adiga, et al.,^26^ India	CS	20/25	60.1 ± 7.8/55.0 ± 10.0	TAS

CS: Case study; 8-oxo-dG: 8-Oxo-2'-deoxyguanosine; MDA: Malondialdehyde; LPO: Lipid hydro-peroxide; NO: Nitric oxide; SOD: Superoxide dismutase; GPx: Glutathione peroxidase; CAT: Catalase; CL-LOOH: Tert-butyl hydroperoxide-initiated chemiluminescence; FOX-LOOH: Lipid hydroperoxides-spectrophotometric assay; TRAP: Total radical-trapping antioxidant parameter; TAS: Total antioxidant status; G6PD: Glucose 6 phosphate dehydrogenase; Ery: Erythrocyte; GSH: Glutathione; TOS: Total oxidant status; OSI: Oxidative stress index; TBARS: Thiobarbituric acid reactive substance

SD: Standard deviation

**Figure 2:**
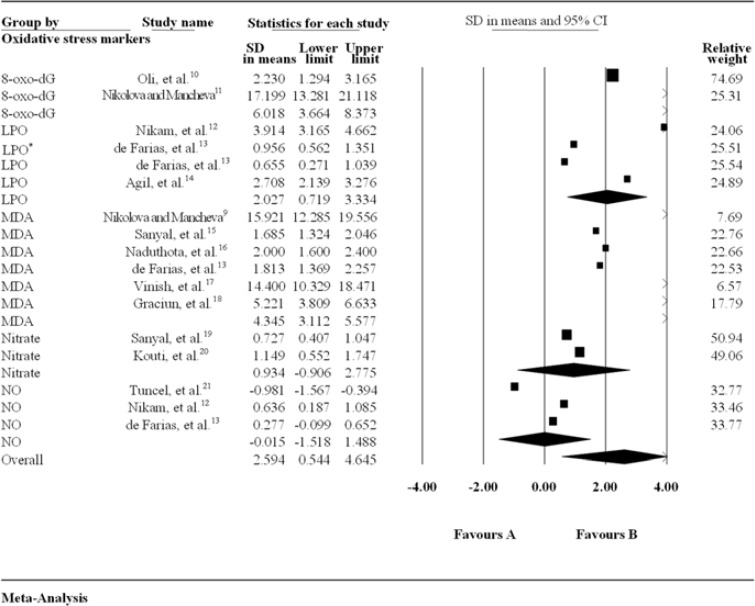
Forest plot of meta-analysis of the relationship between oxidativemarkers and risk of Parkinson’s disease (PD)

SD: Standard difference; 95% CI: 95% of confidence interval; 8-oxo-dG: 8-Oxo-2'-deoxyguanosine; LPO: Lipid hydro-peroxide; MDA: Malondialdehyde; NO: Nitric oxide

^*^The value of LPO was obtained from the two different methods. One value of LPO was obtained from the chemiluminescence assay and other one from spectrophotometer.

**Figure 3:**
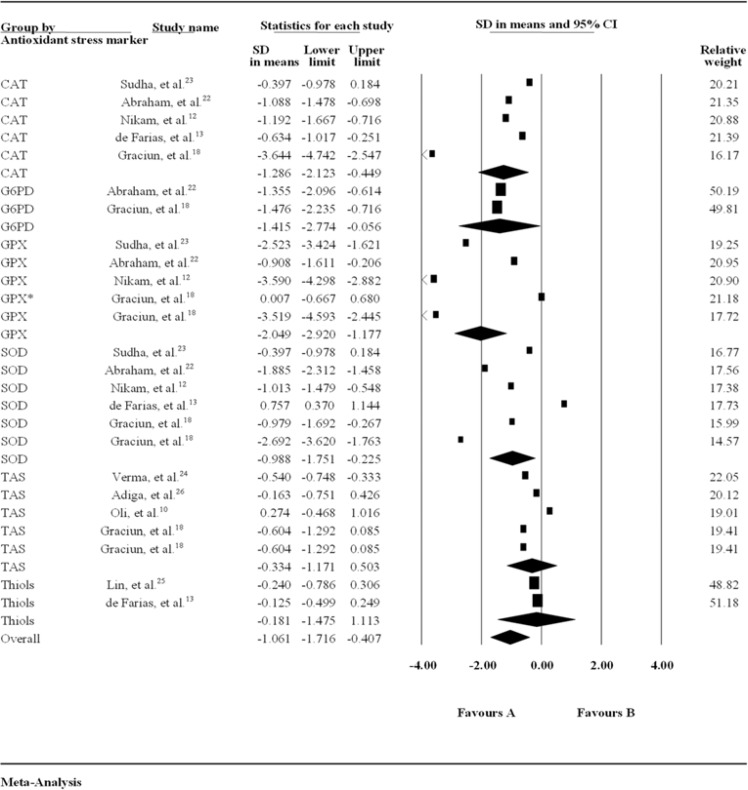
Forest plot of meta-analysis of the relationship between antioxidant markers and risk of Parkinson’s disease (PD)

SD: Standard difference; 95% CI: 95% of confidence interval; CAT: Catalase; G6PD: Glucose 6 phosphate dehydrogenase; GPx: Glutathione peroxidase; SOD: Superoxide dismutase; TAS: Total antioxidant status

^*^The value of LPO was obtained from the two different methods. One value of LPO was obtained from the chemiluminescence assay and other one from spectrophotometer.

Forest plot of SMD + 95% CI of all the biomarkers (Figure 2 and 3) revealed that 3 oxidative stress-related biomarkers namely 8-Oxo-2'-deoxyguanosine (8-oxo-dG), malondialdehyde (MDA), and lipid hydro-peroxide (LPO) showed statistically significant high levels whereas two oxidative stress biomarkers [nitric oxide (NO) and nitrate] did not show statistically significant elevated levels in patients with PD compared to age and sex matched controls. Antioxidant-related biomarkers [SOD, glucose 6 phosphate dehydrogenase (G6PD), glutathione peroxidase (GPx), and CAT] showed statistically significant change, whereas total antioxidant status (TAS) and thiols did not show statistically significant change in patients with PD compared to controls.

***The association between oxidative markers and Risk of PD: ***A forest plot revealed that there was a significant difference obtained for MDA levels (4.345, 95% CI: 3.112-5.577, P < 0.001), 8-oxo-dG levels (6.018, 95% CI: 3.664-8.373, P < 0.001), and LPO levels (2.027, 95% CI: 0.719-3.334, P < 0.001), while there was no significant difference obtained for NO (-0.015, 95% CI: -1.518-1.48, P = 0.980) and nitrate level (0.934, 95% CI: -0.906-2.775, P = 0.400) between PD and control groups. Within oxidative stress biomarkers, high percentage of inconsistency was found to be in 8-oxo-dG (6.018, I^2^ = 98.10%, 95% CI: 3.664-8.373, P < 0.001), and low percentage of inconsistency was obtained in nitrate (0.934, I^2^ = 32.70%, 95% CI: -0.906-2.775, P = 0.300).

***The Relationship between Antioxidant markers and Risk of PD:*** A forest plot revealed that there were a significantly decreased level of CAT activity (1.286, 95% CI: 2.123-0.445, P = 0.003), SOD activity (0.981, 95% CI: 1.751-0.225, P = 0.010), and GPx activity (2.027, 95% CI: 0.719-3.334, P < 0.001); whereas there were no statistically significant `different levels of TAS (0.334, 95% CI: 1.171-0.503, P = 0.400), thiols (0.18, 95% CI: 1.475-1.113, P = 0.700), and G6PD (1.415, 95% CI: 2.774-0.056, P = 0.040) were found between PD and control groups. Within antioxidant biomarkers, high percentage of inconsistency was found to be in SOD (0.981, I^2^ = 95.20%, 95% CI: 1.751-0.225, P < 0.010), and low percentage of inconsistency was obtained in TAS (0.334, I^2^ = 82.24%, 95% CI: 1.171-0.503, P = 0.300). However, due to small sample size and small strata, percentage of inconsistency of thiols and G6PD, analysis has not been possible.

**Table 2:** The relationship between antioxidant markers and risk of Parkinson’s disease (PD)

**Authors**	**Biomarkers**	**Samples ** **(Patients vs. ** **Controls)**	**SMD (95% CI)**	**Heterogeneity**	
				**I** ^2^ ** (%)**	**P**
Sudha, et al.^23^	CAT	15/50	0.397 (0.978-0.184)		
Abraham, et al.^22^	CAT	115/37	1.088 (1.478-0.698)		
Nikam, et al.^12^	CAT	40/40	1.192 (1.667-0.716)		
de Farias, et al.^13^	CAT	56/54	0.634 (1.017-0.251)		
Graciun, et al.^18^	CAT	18/16	3.641 (4.742-2.547)		
SMD (95% CI)		244/197	1.286 (2.123-0.445)	86.90	0.003
Sudha, et al.^23^	SOD	15/50	0.97 (0.978-0.184)		
Abraham, et al.^22^	SOD	115/37	1.885 (2.312-1.458)		
Nikam et al.^12^	SOD	40/40	1.013 (1.479-0.548)		
de Farias et al.^13^	SOD	56/54	0.751 (0.370-0.144)		
Graciun, et al.^18^	SOD from plasma	18/16	0.978 (1.692-0.267)		
Graciun, et al.^18^	SOD from Erythrocytes	18/16	2.691 (3.620-1.762)		
SMD (95% CI)		244/197	0.981 (1.751-0.225)	95.20	0.010
Sudha, et al.^23^	GPx	15/50	2.523 (3.424-1.621)		
Abraham, et al.^22^	GPx	115/37	0.903 (1.611-0.206)		
Nikam, et al.^12^	GPx	40/40	3.590 (4.298-2.882)		
Graciun, et al.^18^	GPx	18/16	3.515 (4.593-2.445)		
SMD (95% CI)		188/143	2.045 (2.920-1.177)	94.30	< 0.001
Abraham, et al.^22^	G6PD	115/37	1.359 (2.099-0.614)		
Graciun, et al.^18^	G6PD	18/16	1.476 (2.235-0.716)		
SMD (95% CI)		133/53	1.415 (2.774-0.056)	SIS	0.041
Verma, et al.^24^	TAS	240/150	0.540 (0.748-0.333)		
Adiga, et al.^26^	TAS	20/25	0.163 (0.751-0.426)		
Oli, et al.^10^	TAS	17/12	0.274 (0.468-1.016)		
Graciun, et al.^18^	TAS	18/16	0.604 (1.292-0.085)		
SMD (95% CI)		295/203	0.334 (1.171-0.503)	82.24	0.400
Lin, et al.^25^	Thiols	27/25	0.240 (0.786-0.306)		
de Farias, et al.^13^	Thiols	56/54	0.123 (0.495-0.249)		
SMD (95% CI)		83/79	0.18 (1.475-1.113)	SIS	0.700

SMD: Standard mean differences; 95% CI: 95% of confidence interval; CAT: Catalase; SOD: Superoxide dismutase; GPx: Glutathione peroxidase; G6PD: Glucose 6 phosphate dehydrogenase; SIS: Substratum is small; TAS: Total antioxidant status

**Publication Bias: **Due to small strata of studies, no significant results obtained from funnel plot. As studies number were small, one-out sensitivity analysis was performed to check the robustness of each marker. The effect size of 8-oxo-dG, CAT, and MDA remained constant after the removal of each study individually.

**Table 3:** The relationship between oxidative markers and risk of Parkinson’s disease (PD)

**Author**	**Biomarkers**	**Samples (Patients ** **vs. Controls)**	**SMD (95% CI)**	**Heterogeneity**	
				**I** ^2^ ** (%)**	**P**
Oli, et al.^10^	8-oxo-dG	17/12	2.23 (1.294-3.165)		
Nikolova and Mancheva^11^	8-oxo-dG	18/20	17.199 (13.281-21.118)		
SMD (95% CI)	8-oxo-dG	35/42	6.018 (3.664-8.373)	98.10	< 0.001
Nikam, et al.^12^	LPO	40/40	3.914 (3.165-4.662)		
de Farias, et al.^13^	LPO*	56/54	0.956 (0.562-1.351)		
de Farias et al.^13^	LPO	56/54	0.655 (0.271-1.039)		
Agil, et al.^14^	LPO	52/40	2.708 (2.139-3.276)		
SMD (95% CI)	LPO	204/188	2.027 (0.719-3.334)	96.30	< 0.001
Nikolova and Mancheva^11^	MDA	18/20	15.921 (12.285-19.556)		
Sanyal, et al.^15^	MDA	80/80	1.685 (1.324-2.046)		
Naduthota, et al.^16^	MDA	72/72	2.000 (1.600-2.400)		
de Farias, et al.^13^	MDA	56/54	1.813 (1.369-2.257)		
Graciun, et al.^18^	MDA	18/16	5.221 (3.809-6.608)		
SMD (95% CI)	MDA	244/242	4.345 (3.112-5.577)	95.60	< 0.001
Sanyal, et al.^19^	Nitrate	80/80	0.727 (0.407-1.047)		
Kouti, et al.^20^	Nitrate	58/15	1.149 (0.552-1.747)		
SMD (95% CI)	Nitrate	138/95	0.934 (-0.906-2.775)	32.70	0.300
Nikam, et al.^12^	NO	40/40	0.636 (0.187-1.085)		
de Farias, et al.^13^	NO	56/54	0.277 (-0.099-0.652)		
Tuncel, et al.^21^	NO	25/25	0.981 (-1.567-0.394)		
SMD (95% CI)	NO	121/119	0.015 (-1.518-1.488)	94.70	0.984

SMD: Standard mean differences; 95% CI: 95% of confidence interval; 8-oxo-dG: 8-Oxo-2'-deoxyguanosine; LPO: Lipid hydro-peroxide; MDA: Malondialdehyde; NO: Nitric oxide

* The value of LPO was obtained from the two different methods. One value of LPO was obtained from the chemiluminescence assay and other one from spectrophotometer.

## Discussion

This systematic literature review aimed to collect, assess, and quantify the relationship between oxidative stress-related biomarkers and patients with PD. Our findings further supported the existence of oxidative damage in these patients. The results showed increased DNA damage in the form of increased level of 8-oxo-dG, high lipid oxidation, and MDA in the patients with PD, while lower activity of scavenging antioxidant enzymes i.e. SOD, CAT, G6PD, and GPx was noticed in these cases as compared to controls.

Oxidative stress is a complex process that involve number of cellular signaling molecules in the form of proteins, enzymes, free radicals, etc. which either increase or decrease at the time of cellular damage.^26^^-^^28^ Oxidative stress can be measured by two ways, first by the biomarkers of oxidative stress, and secondly by calculating antioxidant levels in a cell.^29^^-^^31^ In this meta-analysis, we have found the substantial results as reported by other studies that patients with PD have high oxidative stress as compared to control groups. In our study, nitrate, NO, and TAS found to be non-significantly associated with PD, this might be due to small sample size.

We have tried to calculate the percentage of inconsistency and heterogeneity among studies in this meta-analysis. As strict inclusion criteria have been followed, that includes age- and gender-matched patients with PD and controls along with same diagnostic criteria, due to small study size, the heterogeneity occurred in few biomarkers. This review covered as many as information about the oxidative and antioxidant biomarkers related to PD, but have some limitations also. Patients on DA therapy have been included in this study, the effect of which has not been considered. Positive results with maximum oxidative markers have been observed which might be due to small sample size. Lastly, the study population was mostly from Asians race, generalization of results could not be possible to other populations. To make the studied biomarkers as a gold standard for diagnostic/prognostic purpose, clinical validation will be required.

## Conclusion

Significant association have been observed between oxidative stress and PD. Oxidative stress-related markers as well as signaling pathways can be targeted for therapeutic approach to prevent oxidative stress generation in patients with PD. For the diagnosis of oxidative stress in PD, clinical validation of oxidative stress-related biomarkers would be required for the betterment of these patients.

## Notes:

**How to cite this article:** Khan Z, Ali SA. Oxidative stress-related biomarkers in Parkinson’s disease: A systematic review and meta-analysis. Iran J Neurol 2018; 17(3): 137-44.
